# Artificial Hummingbird Algorithm with Transfer-Learning-Based Mitotic Nuclei Classification on Histopathologic Breast Cancer Images

**DOI:** 10.3390/bioengineering10010087

**Published:** 2023-01-09

**Authors:** Areej A. Malibari, Marwa Obayya, Abdulbaset Gaddah, Amal S. Mehanna, Manar Ahmed Hamza, Mohamed Ibrahim Alsaid, Ishfaq Yaseen, Amgad Atta Abdelmageed

**Affiliations:** 1 Department of Industrial and Systems Engineering, College of Engineering, Princess Nourah bint Abdulrahman University, Riyadh 11671, Saudi Arabia; 2 Department of Biomedical Engineering, College of Engineering, Princess Nourah bint Abdulrahman University, Riyadh 11671, Saudi Arabia; 3 Department of Computer Sciences, College of Computing and Information System, Umm Al-Qura University, Mekkah 24211, Saudi Arabia; 4 Department of Digital Media, Faculty of Computers and Information Technology, Future University in Egypt, New Cairo 11845, Egypt; 5 Department of Computer and Self Development, Preparatory Year Deanship, Prince Sattam bin Abdulaziz University, Al-Kharj 16278, Saudi Arabia

**Keywords:** breast cancer, mitotic nuclei classification, histopathology images, artificial hummingbird algorithm, medical imaging

## Abstract

Recently, artificial intelligence (AI) is an extremely revolutionized domain of medical image processing. Specifically, image segmentation is a task that generally aids in such an improvement. This boost performs great developments in the conversion of AI approaches in the research lab to real medical applications, particularly for computer-aided diagnosis (CAD) and image-guided operation. Mitotic nuclei estimates in breast cancer instances have a prognostic impact on diagnosis of cancer aggressiveness and grading methods. The automated analysis of mitotic nuclei is difficult due to its high similarity with nonmitotic nuclei and heteromorphic form. This study designs an artificial hummingbird algorithm with transfer-learning-based mitotic nuclei classification (AHBATL-MNC) on histopathologic breast cancer images. The goal of the AHBATL-MNC technique lies in the identification of mitotic and nonmitotic nuclei on histopathology images (HIs). For HI segmentation process, the PSPNet model is utilized to identify the candidate mitotic patches. Next, the residual network (ResNet) model is employed as feature extractor, and extreme gradient boosting (XGBoost) model is applied as a classifier. To enhance the classification performance, the parameter tuning of the XGBoost model takes place by making use of the AHBA approach. The simulation values of the AHBATL-MNC system are tested on medical imaging datasets and the outcomes are investigated in distinct measures. The simulation values demonstrate the enhanced outcomes of the AHBATL-MNC method compared to other current approaches.

## 1. Introduction

Mitosis can be defined as a process of cell cycle where a replicated chromosome is split into dual new nuclei that produce genetically identical cells which retain the chromosome number. This method can be split into four phases: telophase, prophase, metaphase, and anaphase. It culminates into two daughter nuclei that are genetically identical [1]. Then, the cell might perform division by cytokinesis to produce dual daughter cells. Producing more than three daughter cells rather than two normal cells is a mitotic fault that might tempt mutations or apoptosis, initiating specific kinds of cancer [2]. In the tissue samples, haematoxylin and eosin (H&E)-stained slides lead to histopathology images where mitosis rate is a significant parameter to determine the tumor aggressiveness, especially breast tumor, and recognition of a typical way of mitosis is utilized as a prognostic and diagnostic marker. Breast tumor is the main factor that leads to higher mortality amongst women and is a frequently diagnosed tumor amongst females; if diagnosed at earlier stages, tit can be the most curable form of tumor [3]. Breast tumor, where survival rate is under 40% in lower-income nations, is the primary tumor type in females globally that costs a great number of lives per annum. As stated by the National Tumor Institution, up to 20% of each breast tumor fails to be found by X-ray mammography (using ionizing radiation) [4]. Mitosis count assists in tumor diagnosis and provides an assessment of tumor aggressiveness that assists in tumor grading. The high number of mitotic cells in a region represents fast-growing or higher-grade tumor.

The visual detection of mitotic nuclei through pathologists is a time-intensive and subjective job with poor reproducibility because of many difficulties. Mitotic nuclei are hyperchromatic objects having different morphological sizes and shapes [5]. Furthermore, the occurrence of mitotic nuclei differs according to tumor stage and tumor grade. In aggressive tumors, generally, mitotic nuclei are nondifferentiable and appear in smaller sizes with higher frequency. The accurate detection of mitotic nuclei depends on the experience and knowledge of the pathologist [6]. Object-level interobserver analysis exposes pathologist disagreement on individual objects. The limitation of manual workflows generates the necessity to automate the count of mitotic nuclei to enhance the decision of the pathologist [7]. For the development of the detection of mitotic nuclei in histopathology images, thus far, various methods have been introduced based on segmentation, classification, and detection methods [8]. The current approaches frequently exploit data balancing methods, namely, rotation, translation, and mirror imaging-oriented techniques for augmenting mitotic examples. Likewise, various researchers implemented a two-step recognition technique to reduce class imbalance and enhance precision [9]. With regard to the complicated nature of mitoses, several research workers used the method of ensemble learning, while few approaches simultaneously trained two deep learning (DL) models to make the concluding decision.

This study designs an artificial hummingbird algorithm with transfer-learning-based mitotic nuclei classification (AHBATL-MNC) on histopathologic breast cancer images. The goal of the AHBATL-MNC technique lies in identification of mitotic and nonmitotic nuclei on histopathology images (HIs). For HI segmentation process, the PSPNet model is utilized to identify the candidate mitotic patches. Next, the residual network (ResNet) model is employed as feature extractor, and the extreme gradient boosting (XGBoost) model is applied as a classifier. To enhance the classification performance, the parameter tuning of the XGBoost model takes place, utilizing the AHBA algorithm. The simulation values of the AHBATL-MNC approach are tested on a medical imaging dataset and the results are investigated in distinct measures.

## 2. Related Works

Shwetha and Dharmanna [10] modeled a new technique for automatic identification and detection by DL model. In this presented technique, the work can be split into five phases. In the initial phase, histopathological images are preprocessed to boost the contrast of the nonmitotic and mitotic cells through image adjustment method. In the next phase, using Otsu segmentation method, the background and foreground are divided. In [11], the author devised a new structure called SmallMitosis for identifying mitotic cells that are very small in size undergoing mitosis out of the H&E-stained breast histological images. SmallMitosis structure has a deep multiscale (MS-RCNN) detector and an atrous fully convolution-oriented segmentation (A-FCN) method. In the A-FCN technique, the atrous convolution concept aids in predict bounding box annotations and mitosis masks of very-small-sized mitotic cells.

Sohail et al. [12] devised an innovative deep convolutional neural network (DCNN)-related heterogeneous ensemble method, “DHE-Mit-Classifier”, for examining mitotic nuclei in breast histopathological imageries. Sebai et al. [13] proposed an accurate and robust algorithm for detecting mitoses automatically from histology breast cancer slides by making use of the multitask DL structure for instance segmentation mask region-based convolutional neural network (RCNN) and object detection. Lei et al. [14] devised an accurate and fast approach to automatically identify mitosis from histopathology images. This presented algorithm is capable of detecting the mitotic candidates automatically from histological units for mitosis screening. In particular, this technique uses DCNN for extracting high-level features of mitosis to find mitotic applicants. After that, the author employed spatial attention elements to re-encode mitotic features that enabled the method to very effectively study features. 

Das and Dutta [15] introduced an innovative technique for mitotic cell recognition in breast histology images, exploiting wavelet decomposed image patches and DCNN. In this method, Haar wavelet is used to formulate a DCNN technique for automatic recognition of mitotic cells. The decomposition step reduces convolutional period for mitotic cell recognition related to the usage of raw image patches in traditional DCNN approaches. Beevi et al. [16] explored the feasibility of transfer learning (TL) for mitosis recognition. A pretrained convolutional neural network (CNN) was shown by merging RF method with the initial FC layers for deriving discriminant features from nuclei patches and to accurately prognosticate class labels of cell nuclei. The altered CNN precisely categorizes the identified cell nuclei with limited trained datasets. This structure would establish maximum classifier accuracy by prudently preprocessing the extracted features and fine-tuning the pretrained methods. 

## 3. The Proposed Mitotic Nuclei Classification Model

**Figure 1:**
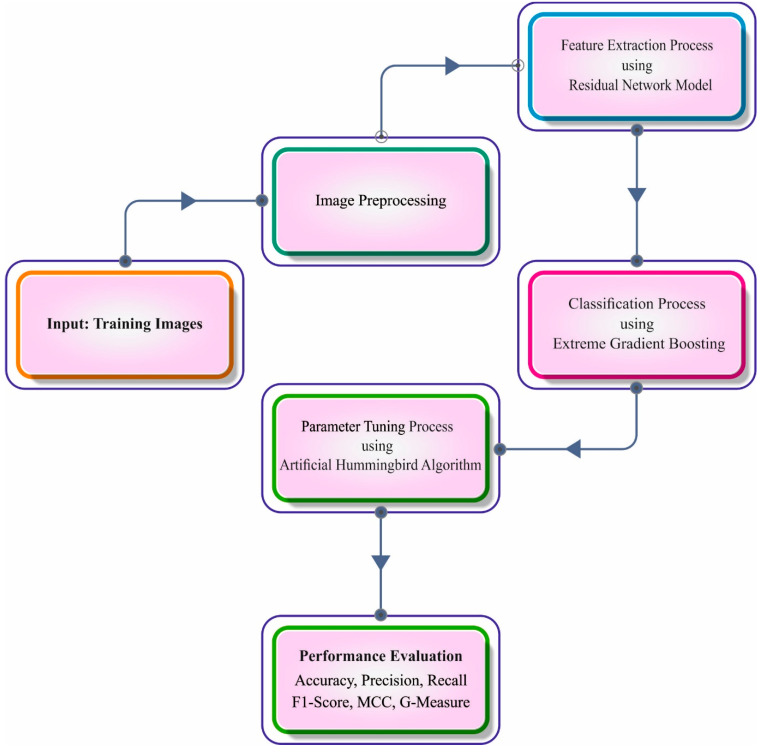
Overall working process of AHBATL-MNC system.

In this study, we develop a new AHBATL-MNC technique for effective identification of mitotic and nonmitotic nuclei on HIs. The presented AHBATL-MNC technique encompasses a series of processes, namely, PSPNet segmentation, ResNet feature extraction, XGBoost classification, and AHBA parameter tuning. Figure 1 defines the overall work flow of the AHBATL-MNC system.

### 3.1. Segmentation Process

In the AHBATL-MNC technique, the PSPNet model is utilized for segmentation process. PSPNet is the renowned network architecture for semantic segmentations [17]. The PSPNet was initially introduced for scene parsing. To aggregate multiscale contextual datasets, one pyramid pooling network (PPM) was introduced in PSPNet. At first, max pooling is enforced to generate a feature map using three pyramid scales that can be attained by Equation (1), wherein FDS and λ, correspondingly, signify input and downsampling method through max pooling, and stride of max pooling layer can also be attained using Equation (2):

(1) $$F_{j}=DS(F,\lambda_{j}.)j=1,2,3$$

(2) $$\frac{w-\lambda_{j}}{\lambda_{j}}+1=0_{j}\Rightarrow \lambda_{j}=\frac{w}{o_{j}}$$

whereas w and 0 signify input and output size of feature maps.

After applying convolution method to these multiscale feature maps, bilinear interpolation can be performed to resize feature maps, whereas $W_{j}^{T}$ and $b_{j}$, correspondingly, denote the weight and bias of j-th 1×1 convolutional layer, and BI(.) denotes the bilinear interpolation.

(3) $$O_{j}=BI\left(W_{j}^{T}⊗F_{j}+b_{j}\right)j=1,2,3$$

Likewise, the feature maps having the new input and pyramid scale were concatenated, and 1×1 convolution was implemented to reduce channel number of output, whereas $W_{j}^{T}$ and $b_{j}$ demonstrate weight and bias of the 1×1 convolution layer.

(4) $$C=W_{rd}^{T}\left(concat\left(F,O_{1},O_{2},O_{3}\right)\right)+b_{rd}$$

Dissimilar to the original PPM, feature maps having four pyramid scales, which include 1, 2, 3, and 6, are constructed by the new PPM, whereas feature maps having three pyramid scales, including 1, 2, and 6, are constructed by max pooling.

Furthermore, the 1×1 convolution layer is interconnected with the concatenation layer for dimensionality reduction.

Based on the UNet structure, a multilevel PSPNet is introduced as the decoder. The 1, 2, and 3 attention gates are enforced to correspondingly generate initial convolutional layer and the attention maps of third and fifth identity blocks. In addition, to incorporate multilevel features, the attention gate and the output of PPM are concatenated densely with the following equation:

(5) $$Y_{j}=concat\left(US\left(C_{j},3\right)M_{-}output_{j}\right)j=1,2,3$$

### 3.2. Feature Extraction Process

In this study, the ResNet model was employed as feature extractor. We adapted the CNN, ResNet50, to characterize the image, and the deep network has 50 layers [18]. The depth of network was crucial for neural network (NN), but a deep network can be tough to train. The ResNet50 infrastructure facilitates the network training and permits it to be deeper which leads to enhanced efficacy in diverse tasks. ResNet50 is deeper than simple counterparts, but parameter count of these networks is smaller. A DCNN resulted in a series of breakthroughs for image classification. Many nontrivial visual detection techniques have benefitted from deep methods. Once the network depth rises, performance of the network degrades quickly (saturated) and rapidly increases. Meanwhile, deep networks have large representation power. It can be possible for ResNet50 to accomplish a deep model that is not worse than lesser deep networks. It is implemented by adding numerous identity layers, viz., levels that skip signal without further amendment. ResNet50 deep level has to predict variations amongst the main function and outcome of the previous layer.

The method considers the image and generates the caption, encrypted as a series of 1-K codewords.

(6) $$y=\left\{y_{1},y_{2},\cdots ,y_{c}\right\},y_{j}\in R^{K}$$

From the expression, K indicates the dictionary size and c represents caption length. The extractor will produce L-vectors, each having D-dimensional representation of the image.

The hyperparameter tuning of the ResNet model is performed by the Adamax optimizer [19]. It is an amended form of the Adam optimizer where the distributed variance is projected ∞. In addition, the maximized weight can be determined as follows:

(7) $$w_{t}^{i}=w_{t-1}^{i}-\frac{\eta }{v_{t}+\epsilon }\times {\hat{m}}_{t}$$

whereas

(8) $${\hat{m}}_{t}=\frac{m_{t}}{1-\beta_{1}^{t}}$$

(9) $$v_{t}=max(\beta_{2}\times v_{t-1},|G_{t}|)$$

(10) $$m_{t}=\beta_{1}m_{t-1}+(1-\beta_{1})G$$

(11) $$G=\nabla_{w}C\left(w_{t}\right)$$

From the expression, η denotes the rate of learning, $w_{t}$ refers to the weights at steps t, $C\left(.\right)$ signifies the cost function, and $\nabla_{w}C\left(w_{t}\right)$ suggests the gradient of weight variable $w_{t}$ x and equal label y. $\beta_{i}$ is employed to select the data needed for older upgrades, when $\beta_{i}\in [0,1].$ $m_{t}$ and $v_{t}$ are the first and second moments as explained in Algorithm 1.
**Algorithm 1:** Pseudocode of Adamaxη: Rate of Learning $\beta_{1}$, $\beta_{2}\in$ [0, 1): Exponential decomposed values to moment candidate $C\left(w\right)$: Cost function with w variable $w_{0}$: parameter vector $m_{0}\leftarrow 0$ $u_{0}\leftarrow 0$ i←0 (Implement time step) while w does not converge do              i←i+1       $m_{i}\leftarrow \beta_{1}\times m_{i-1}+(1-\beta_{1})\times \frac{\partial C}{\partial w}\left(w_{i}\right)$         $u_{i}\leftarrow max\left(\beta_{2}\times u_{i-1},\left|\frac{\partial C}{\partial w}\left(w_{i}\right)\right|\right)$ $w_{i+1}\leftarrow w_{i}-(\eta /(1-\beta_{1}^{i}))\times m_{i}/u_{i}$ end while show $w_{i}$ (end parameter)

### 3.3. Optimal Classification Process

Finally, the XGBoost model is exploited for classification purposes. XGBoost is used to classify the regression tree model that comes from the gradient lifting decision tree (DT) [20]. The presented algorithm is used for the pedestrian detection classifier. Firstly, it learns a tree from a sample to attain the initial assessment outcome Y1, and then learns with y based on the variance between the predictive and the real labels in the prior step. Likewise, the model error can be reduced successfully. Equations (4)–(8) provide the assessment flow of XGBoost training. The subsequent formula is to evaluate the target of n-th tree models. The primary behavior determines a regularization term that could decrease overfitting to enhance the generalization ability. Taylor’s expansion has first and second derivatives and constant terms.

Among them, the objective function of every round is evaluated as follows, and $f_{t}$ can be selected for minimizing the main function, viz., the error between actual outcome and the predictive outcome is decreased after adding $f_{t}$. Here, l represents the error function and Ω denotes a regularization term, the error function tries to fit the training dataset, and the regularization term encourages a simple method. The randomness of the outcomes of the finite data fitting is very small, which is not easy for overfitting, making the prediction of the concluding model more stable.

(12) $$Obj^{\left(t\right)}=\sum_{i=1}^{n}l\left(y_{i},{\hat{y}}_{i}^{\left(t-1\right)}+f_{t}\left(x_{i}\right)\right)+\Omega \left(f_{i}\right)+constant$$

Once the error function l is not a square error, the first three terms of the Taylor equation are utilized for approximating original objective function.

(13) $$Obj^{\left(t\right)}=\sum_{i=1}^{n}[l(y_{i},{\hat{y}}^{\left(t-1\right)})+g_{i}f_{t}(x_{t})+\frac{1}{2}h_{l}f_{t}^{2}(x_{i})]+\Omega (f_{t})+constant$$

where $g_{i}$ and $h_{i}$ refer to the initial and second derivatives of the error function.

(14) $$g_{i}=\partial_{{\hat{y}}_{i}^{\left(t-1\right)}}l(y_{i},{\hat{y}}^{\left(t-1\right)})$$

(15) $$h_{i}=\partial_{{\hat{y}}_{i}^{\left(t-1\right)}}^{2}l(y_{i},{\hat{y}}_{i}^{\left(t-1\right)})$$

Next, we eliminate the constant terms, such as the variance between real value and predicted value of the previous round.

(16) $$Obj^{\left(t\right)}=\sum_{i=1}^{n}[g_{l}f_{t}(x_{i})+\frac{1}{2}h_{\iota }f_{t}^{2}(x_{i})]+\Omega (f_{t})$$

According to the realization of XGBoost, the model initially ranks the eigenvalue, since the tree model should define the better segmentation points and later store them in numbers of blocks. This architecture is reutilized in later iterations, which significantly decreases the computation difficulty. Furthermore, the data gain of every feature should be evaluated in the procedure of node splitting, hence the computation of data gain is parallelized through the data structure.

For improving the classification performance, the parameter tuning of the XGBoost model is performed by the AHBA technique. AHBA is a population-related metaheuristic approach that primarily simulates three foraging behaviors of hummingbirds (HB): migratory, guided, and territorial foraging [21]. In the foraging process, the three flight skills include axial, diagonal, and modeled-omnidirectional flights. Simultaneously, an access table simulating HB remarkable memory capability is created for guiding HB to carry out global optimization. The three flying skills are described in the following: the flight skill simulation is expanded to d-D space with axial flight and can be given in Equation (17):

(17) $$D^{\left(i\right)}=\left\{\begin{array}{ll} 1 & \mathrm{if}\;i=randi([1,d])i=1,\cdots ,d\\ 0 & \;\;\;\;\;\;\;\;\;\;\;\;\;\;\;\;\;\;\;\;\;\;else \end{array}\right.$$

Diagonal flight can be determined by Equation (18):

(18) $$D^{\left(i\right)}=\left\{\begin{array}{l} 1,if\;i=p(j)P=randperm(k),k\in [2,\lceil r_{1}(d-2)\rceil +1]\\ 0,else. \end{array}\right.$$

Omnidirectional flight is defined below:

(19) $$D^{\left(i\right)}=1i=1,\cdots ,d$$

In Equation (19), randi([1,d]) creates a random number from 1 to d, randperm(k) generates a random permutation of integer from 1 to k, and $r_{1}$ indicates a random integer that ranges from zero to one. First, the AHA initializes a visiting table and a set of random solutions. In all the iterations, territorial or guided foraging can be carried out 50% of the time. Hummingbirds move toward the food sources using guided foraging, viz., depending on a visiting table and nectar filling rate. Territorial foraging enables HBs to find new food sources as candidates and easily move toward the neighboring region within their territory. Migration foraging can be performed in each of two iterations. Until the stopping condition is met, each operation and calculation are interactively performed. At last, the food source with the maximum rate of nectar refilling is returned as near-global optimal.

(1) A population of n HBs is initialized at random to n food source in the following:
  (20) $$\chi_{j}=Low+r\times (Up-Low)i=1,\cdots \cdots n$$

In Equation (20), Low and Up indicate the lower and upper limitations for d-dimension problems, correspondingly; r refers to a random integer within the range of zero and one; $x_{i}$ signifies the location of the i-th food sources.

(21) $$VT_{i,j}=\left\{\begin{array}{ll} 0 & ifi\neq j\\ null & i=j \end{array}\right.$$

where i=j, $VT_{i,j}=null$ shows that an HB takes food from a certain food source; i≠j, $VT_{i,j}=0$ denotes that the j-th food sources were visited by i-th HB in the present iteration.

(2) Guided foraging: With the abovementioned flight abilities, an HB could access its targeted food sources to attain candidate food source, hence the following mathematical expression simulates candidate food source and guiding foraging behaviors:
  (22) $$v_{i}\left(t+1\right)=x_{i,tar}\left(t\right)+a\times D\times (x_{i}\left(t\right)-x_{i,tar}\left(t\right))$$
  (23) $$a∼N\left(0,1\right)$$

From the expression, $x_{i}(t)$ and $x_{i,tar}(t)$ are the position of i-th hummingbird food and target source at t time; a is distributed uniformly, with standard deviation of 1 and mean =0.

The location updating of i-th food sources is given below:

(24) $$x_{i}\left(t+1\right)=\left\{\begin{array}{ll} \chi_{i(t)} & f(\chi_{i}(t))\leq f(v_{j}(t+1))\\ v_{i}(t+1) & f(x_{i}(t))>f(v_{i}(t+1)) \end{array}\right.$$

In Equation (24), f(·) denotes function fitness value. Equation (24) represents that if the nectar refilling rate of candidate food sources is superior to the present one, the HB will abandon the existing food source and stay at a candidate one for feeding.

(3) Territorial foraging: After attaining targeted food sources where nectar was eaten, an HB seeks innovative food sources. Thus, an HB could move towards a neighboring region within its own territory whereby a novel food source is found that is the best candidate solution. The mathematical expression to stimulate local search of an HB for territorial foraging strategy and candidate food source is shown below:
  (25) $$v_{i}\left(t+1\right)=x_{i}\left(t\right)+b\times D\times x_{i}\left(t\right)$$
  (26) $$b∼N\left(0,1\right)$$

Now, b is distributed uniformly, with a standard deviation of 1 and mean = 0.

(4) Once food becomes frequently scarce in a territory visited by an HB, the bird frequently migrates to more distant food sources for foraging.

## 4. Results and Discussion

**Figure 2:**
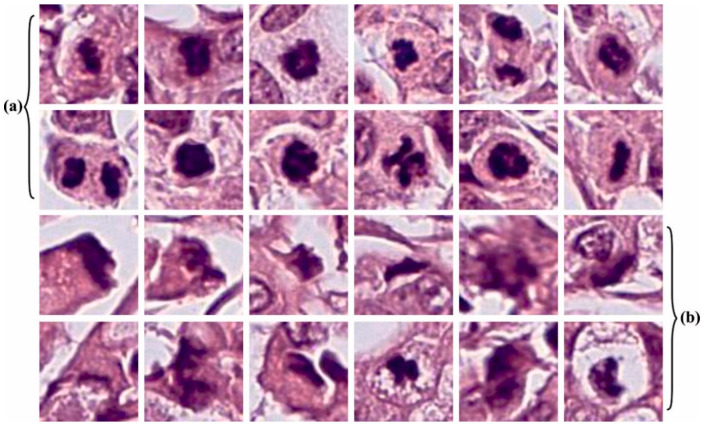
Sample images of (**a**) mitosis; (**b**) nonmitosis.

The proposed model is simulated using Python 3.6.5 tool on PC i5-8600k, GeForce 1050Ti 4 GB, 16 GB RAM, 250 GB SSD, and 1 TB HDD. The parameter settings are given as follows: learning rate: 0.01, dropout: 0.5, batch size: 5, epoch count: 50, and activation: ReLU. The experimental validation of the AHBATL-MNC method on mitosis cell classification is tested using a dataset [22] that has 150 images and two classes, as represented in Table 1. Figure 2 depicts some sample images of mitosis and nonmitosis.

**Figure 3:**
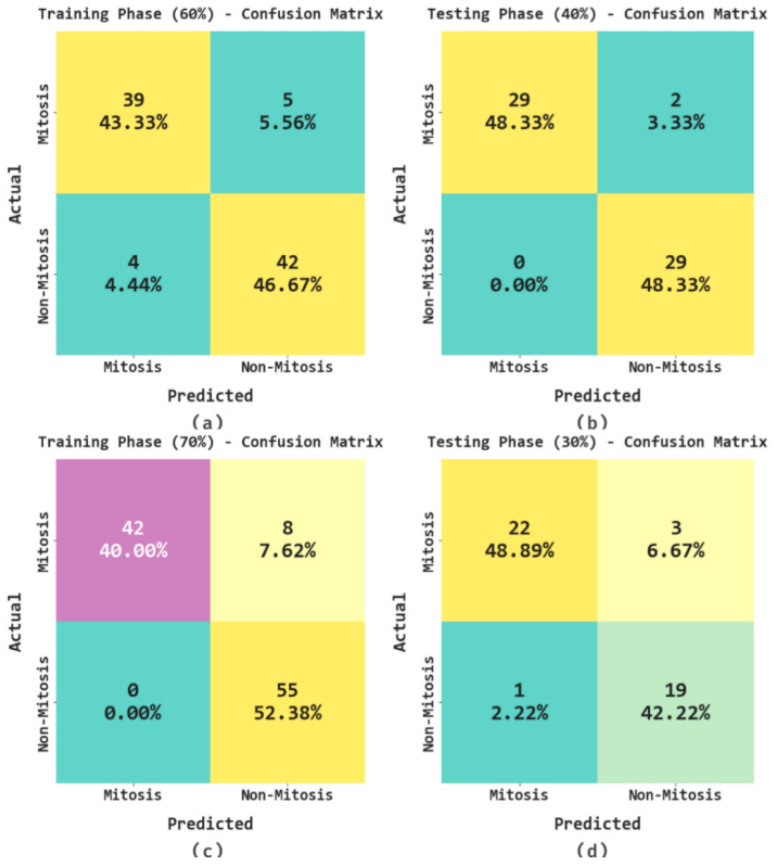
Confusion matrices of AHBATL-MNC system. (**a**,**b**) TR and TS databases of 60:40; (**c**,**d**) TR and TS databases of 70:30.

The binary classification outcomes of the AHBATL-MNC method on the applied dataset are portrayed in the form of confusion matrix in Figure 3. On 60% of the training (TR) database, the AHBATL-MNC model detected 39 samples into mitosis class and 42 samples into nonmitosis class. Meanwhile, on 40% of the testing (TS) database, the AHBATL-MNC method detected 29 samples into mitosis class and 29 samples into nonmitosis class. Eventually, on 70% of the TR database, the AHBATL-MNC system detected 42 samples into mitosis class and 55 samples into nonmitosis class. Finally, on 30% of the TS database, the AHBATL-MNC algorithm detected 22 samples into mitosis class and 19 samples into nonmitosis class.

**Figure 4:**
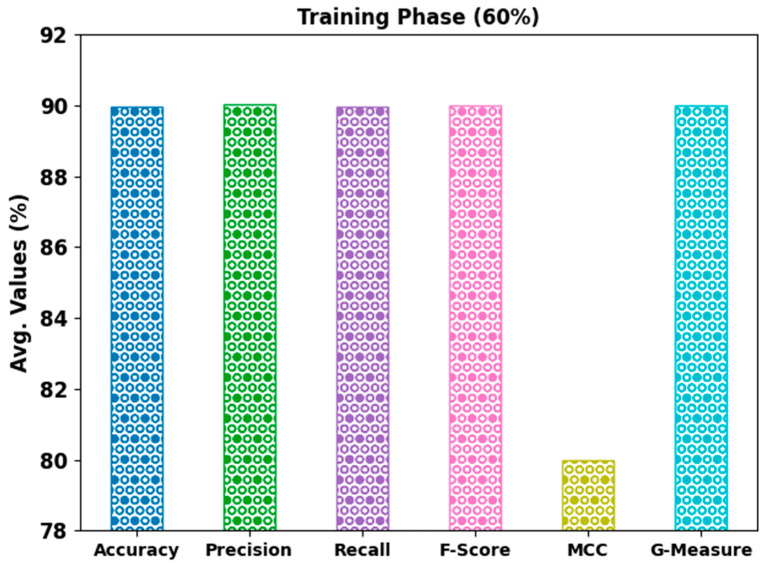
Average analysis of AHBATL-MNC approach under 60% of TR database.

In Table 2, overall mitosis classification results of the AHBATL-MNC model under 60% of TR and 40% of TS databases are given. Figure 4 exhibits the detailed classifier outcome of the AHBATL-MNC model on 60% of the TR database. The outcomes depict that the AHBATL-MNC model properly classified mitosis and nonmitosis class images. It is noted that the AHBATL-MNC model attained average $accu_{bal}$ of 89.97%, $prec_{n}$ of 90.03%, $reca_{l}$ of 89.93%, $F_{score}$ of 89.99%, MCC of 80%, and $G_{measure}$ of 89.99%.

**Figure 5:**
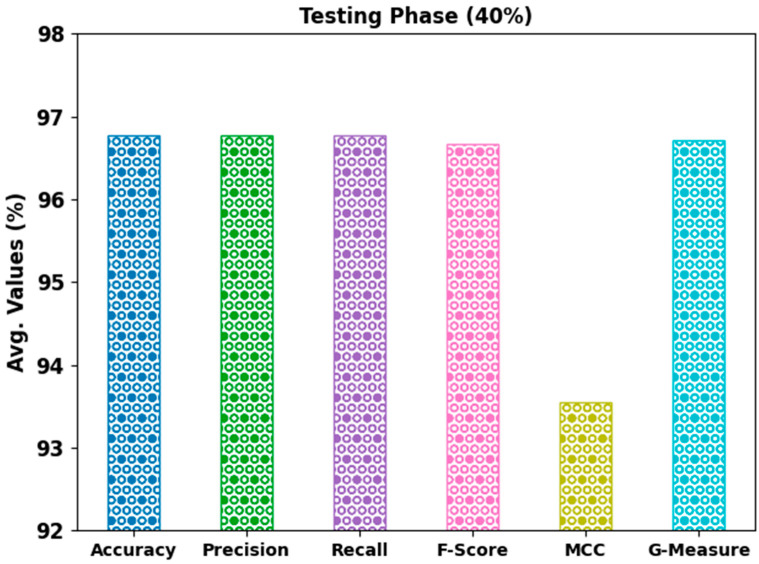
Average analysis of AHBATL-MNC approach under 40% of TS database.

Figure 5 reveals a comprehensive classifier outcome of the AHBATL-MNC system on 40% of the TS database. The outcomes show that the AHBATL-MNC approach properly classified the mitosis and nonmitosis class images. It can be seen that the AHBATL-MNC method reached average $accu_{bal}$ of 96.77%, $prec_{n}$ of 96.77%, $reca_{l}$ of 96.77%, $F_{score}$ of 96.67%, MCC of 93.55%, and $G_{measure}$ of 96.72%.

**Figure 6:**
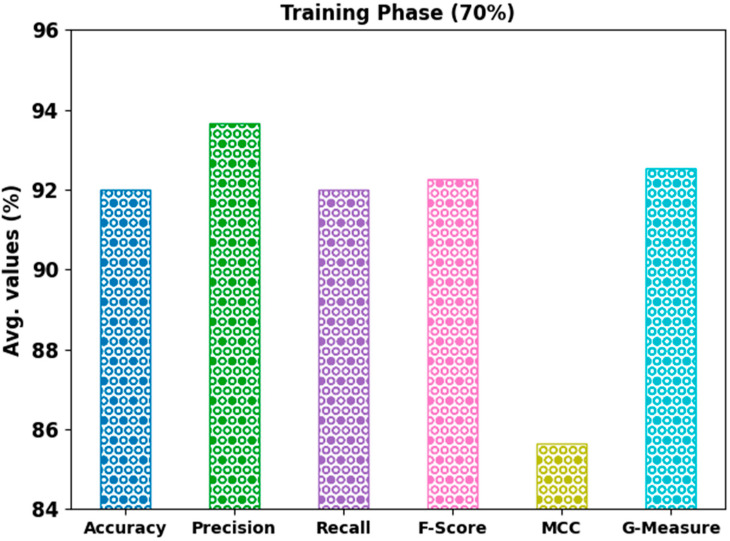
Average analysis of AHBATL-MNC approach under 70% of TR database.

In Table 3, the overall mitosis classification outcome of the AHBATL-MNC algorithm under 70% of the TR and 30% of the TS databases is given. Figure 6 demonstrates the detailed classifier outcome of the AHBATL-MNC method on 70% of the TR database. The outcomes represent that the AHBATL-MNC system properly classified the mitosis and nonmitosis class images. It is clear that the AHBATL-MNC methodology obtained average $accu_{bal}$ of 92%, $prec_{n}$ of 93.65%, $reca_{l}$ of 92%, $F_{score}$ of 92.26%, MCC of 85.63%, and $G_{measure}$ of 92.54%.

**Figure 7:**
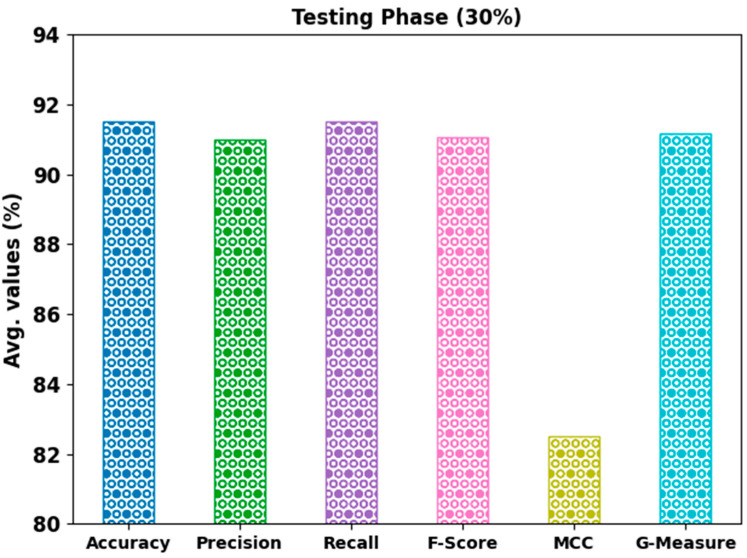
Average analysis of AHBATL-MNC approach under 30% of TS database.

Figure 7 shows a brief classifier outcome of the AHBATL-MNC approach on 30% of the TS database. The outcome demonstrates that the AHBATL-MNC algorithm properly classified the mitosis and nonmitosis class images. It can be stated that the AHBATL-MNC algorithm accomplished average $accu_{bal}$ of 91.50%, $prec_{n}$ of 91.01%, $reca_{l}$ of 91.50%, $F_{score}$ of 91.07%, MCC of 82.51%, and $G_{measure}$ of 91.16%.

**Figure 8:**
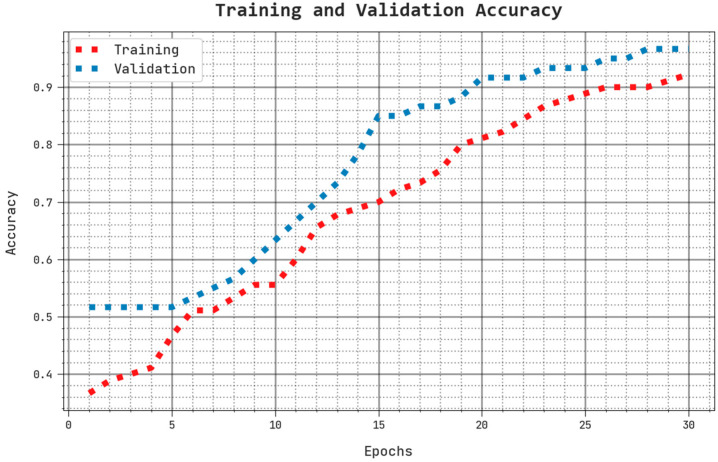
TACC and VACC analysis of AHBATL-MNC approach.

The training accuracy (TACC) and validation accuracy (VACC) of the AHBATL-MNC system are inspected on breast cancer performance in Figure 8. The figure reveals that the AHBATL-MNC approach shows improved performance with improved values of TACC and VACC. It is noticeable that the AHBATL-MNC system gained higher TACC outcomes.

**Figure 9:**
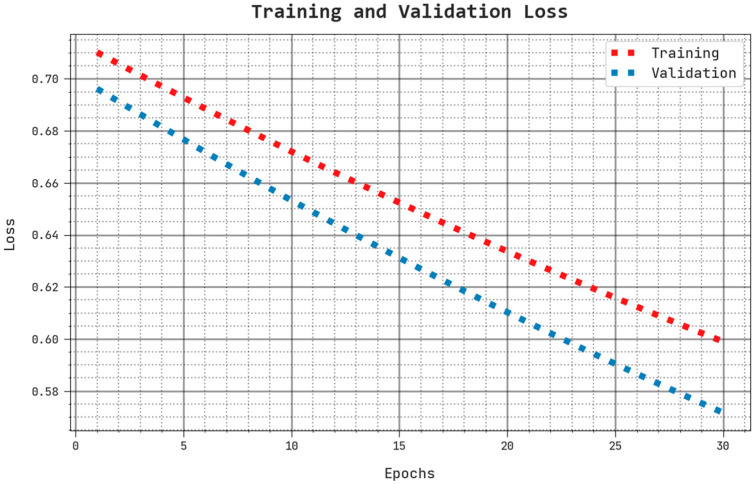
TLS and VLS analysis of AHBATL-MNC approach.

The training loss (TLS) and validation loss (VLS) of the AHBATL-MNC methodology are tested on breast cancer performance in Figure 9. The figure points out that the AHBATL-MNC algorithm revealed better performance with lower values of TLS and VLS. It is observable that the AHBATL-MNC methodology resulted in minimal VLS outcomes.

**Figure 10:**
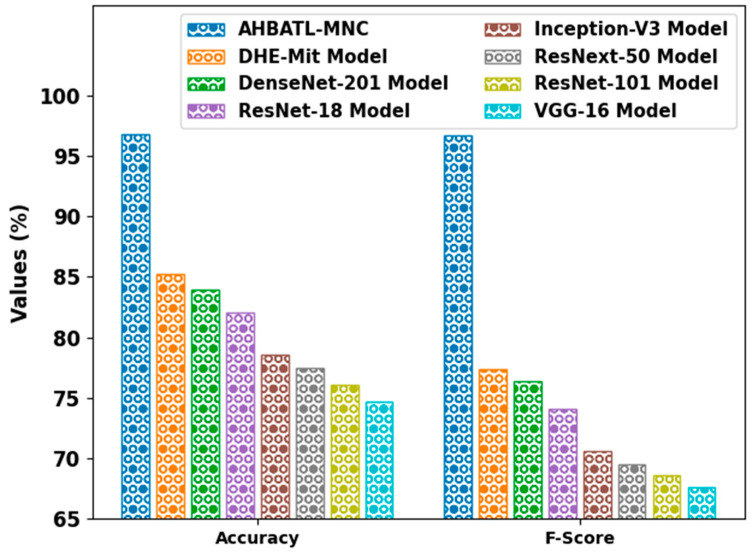
*Accu_y_* and *F_score_* analysis of AHBATL-MNC system compared with other approaches.

Table 4 reports an overall comparative inspection of the AHBATL-MNC method with recent approaches [13]. Figure 10 offers a comparative inspection of the AHBATL-MNC method in terms of $accu_{y}$ and $F_{score}$. The outcomes indicate that the AHBATL-MNC method achieved improved performance. For instance, based on $accu_{y}$, the AHBATL-MNC model obtained higher $accu_{y}$ of 96.77%. In contrast, the DHE-Mit, DenseNet-201, and ResNet-18 models attained lower $accu_{y}$ of 85.23%, 83.96%, and 82.01%, respectively. Eventually, with respect to $F_{score}$, the AHBATL-MNC approach gained maximal $F_{score}$ of 96.67%. In contrast, the DHE-Mit, DenseNet-201, and ResNet-18 systems obtained decreased $F_{score}$ of 77.33%, 76.38%, and 74.05%, correspondingly.

**Figure 11:**
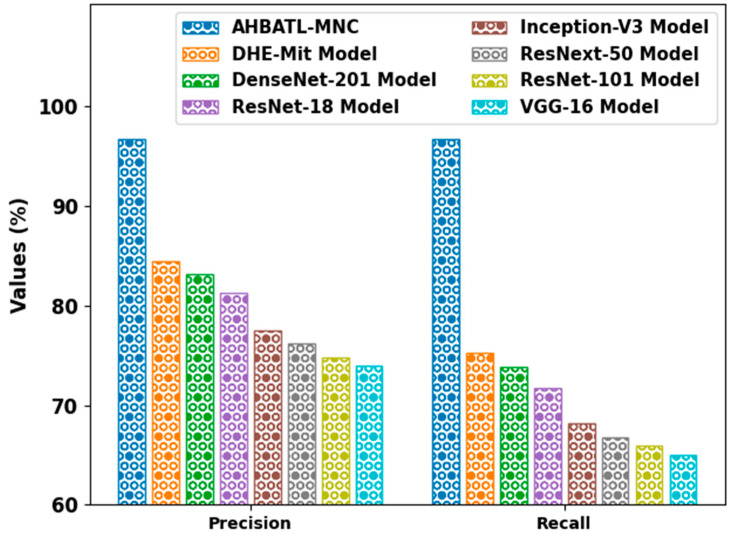
*Prec_n_* and *Reca_l_* analysis of AHBATL-MNC system compared with other approaches.

Figure 11 provides a comparative examination of the AHBATL-MNC approach with respect to $prec_{n}$ and $reca_{l}$. The outcomes state that the AHBATL-MNC approach gained enhanced performance. For example, in terms of $prec_{n}$, the AHBATL-MNC model obtained higher $prec_{n}$ of 96.77%. In contrast, the DHE-Mit, DenseNet-201, and ResNet-18 models attained lower $prec_{n}$ of 84.45%, 83.20%, and 81.26%, correspondingly. Finally, with respect to $reca_{l}$, the AHBATL-MNC model gained enhanced $reca_{l}$ of 96.77%. In contrast, the DHE-Mit, DenseNet-201, and ResNet-18 methods accomplished lower $reca_{l}$ of 75.26%, 73.85%, and 71.73%, respectively.

Table 5 offers a detailed computation time (CT) examination of the proposed model with existing models. The experimental results indicate that the proposed model shows better performance with minimum CT of 12.34 s. On the contrary, the existing models attained increased CT values compared to the AHBATL-MNC model. These results confirm the improvement of the AHBATL-MNC model over other models. The proposed model accomplished superior performance to other existing techniques due to the hyperparameter selection of ResNet using Adamax optimizer and AHBA for XGBoost classifier.

## 5. Conclusions

In this study, we developed a new AHBATL-MNC technique for effective identification of mitotic and nonmitotic nuclei on His. Primarily, in the AHBATL-MNC technique, the PSPNet model is utilized for segmentation process, which identifies the candidate mitotic patches. Followed by this, the ResNet model is employed as feature extractor, and the XGBoost model is applied as a classifier. For improving the classification performance, the parameter tuning of the XGBoost model was performed by the AHBA technique. The performance evaluation of the AHBATL-MNC technique was tested on medical imaging datasets and the outcomes were examined in distinct measures. The simulation values validated the improved outcomes of the AHBATL-MNC algorithm over other recent approaches. In future, the performance of the AHBATL-MNC method can be improved by the use of ensemble learning methodologies. In addition, the proposed model needs to be tested on large-scale databases and can be extended to detect other kinds of cancer.

## Figures and Tables

**Table 1:** Dataset details.

Class	No. of Images
Mitosis	75
Nonmitosis	75
Total Number of Images	150

**Table 2:** Mitosis classification outcome of AHBATL-MNC approach under 60:40 of TR/TS databases.

Class	Accuracy_bal_	Precision	Recall	F-Score	MCC	G-Measure
Training Phase (60%)						
Mitosis	88.64	90.70	88.64	89.66	80.00	89.66
Nonmitosis	91.30	89.36	91.30	90.32	80.00	90.33
Average	89.97	90.03	89.97	89.99	80.00	89.99
Testing Phase (40%)						
Mitosis	93.55	100.00	93.55	96.67	93.55	96.72
Nonmitosis	100.00	93.55	100.00	96.67	93.55	96.72
Average	96.77	96.77	96.77	96.67	93.55	96.72

**Table 3:** Mitosis classification outcome of AHBATL-MNC approach under 60:40 of TR/TS databases.

Class	Accuracy_bal_	Precision	Recall	F-Score	MCC	G-Measure
Training Phase (70%)						
Mitosis	84.00	100.00	84.00	91.30	85.63	91.65
Nonmitosis	100.00	87.30	100.00	93.22	85.63	93.44
Average	92.00	93.65	92.00	92.26	85.63	92.54
Testing Phase (30%)						
Mitosis	88.00	95.65	88.00	91.67	82.51	91.75
Nonmitosis	95.00	86.36	95.00	90.48	82.51	90.58
Average	91.50	91.01	91.50	91.07	82.51	91.16

**Table 4:** Comparative analysis of AHBATL-MNC system with other approaches.

Methods	$accu_{y}$	$prec_{n}$	$reca_{l}$	$F_{score}$
AHBATL-MNC	96.77	96.77	96.77	96.67
DHE-Mit model	85.23	84.45	75.26	77.33
DenseNet-201 model	83.96	83.20	73.85	76.38
ResNet-18 model	82.01	81.26	71.73	74.05
Inception-V3 model	78.54	77.51	68.18	70.64
ResNext-50 model	77.48	76.20	66.73	69.49
ResNet-101 model	76.03	74.83	65.89	68.65
VGG-16 model	74.72	73.93	65.00	67.66

**Table 5:** Comparative CT analysis of AHBATL-MNC system with other approaches.

Methods	Computational Time (s)
AHBATL-MNC	12.34
DHE-Mit model	25.17
DenseNet-201 model	42.58
ResNet-18 model	41.03
Inception-V3 model	59.67
ResNext-50 model	39.36
ResNet-101 model	44.60
VGG-16 model	56.14

## Data Availability

Data sharing not applicable to this article as no datasets were generated during the current study.
